# Machine learning driven precision medicine in tacrolimus dosing: current research and future perspectives in liver and kidney transplantation

**DOI:** 10.3389/fimmu.2026.1822904

**Published:** 2026-07-20

**Authors:** Hanbei Lv, Zhangpeng Feng, Shusen Zheng, Guoping Jiang

**Affiliations:** 1 School of Medicine, Zhejiang Chinese Medical University, Hangzhou, China; 2 Department of Hepatobiliary Pancreatic Surgery, Key Laboratory of Artificial Organs and Computational Medicine in Zhejiang Province, Shulan (Hangzhou) Hospital, Shulan International Medical College, Zhejiang Shuren University, Hangzhou, China; 3 Department of Hepatobiliary Pancreatic Surgery, Shulan (Hangzhou) Hospital, Shulan International Medical College, Zhejiang Shuren University, Hangzhou, China

**Keywords:** blood concentration prediction, dynamic dose optimization, individualized treatment, machine learning, organ transplantation, tacrolimus

## Abstract

**Background:**

Tacrolimus (TAC) is a core immunosuppressant used to prevent transplant rejection after organ transplantation. However, its clinical use is limited by a narrow therapeutic window and substantial interindividual pharmacokinetic variability. Subtherapeutic TAC concentrations are closely associated with acute or chronic transplant rejection, whereas supratherapeutic concentrations often cause severe drug toxicity. As precision medicine advances, integrating multidimensional patient clinical characteristics with machine learning (ML) provides important support for individualized TAC dosing adjustments after liver and kidney transplantation.

**Aim:**

This review summarizes the application of ML in determining the initial dose of TAC and in recommending early blood drug concentrations in liver and kidney transplant patients, analyzes the core challenges in model extrapolation and clinical translation in existing research, compares their characteristics with traditional models, and looks ahead to the construction direction of long-term immunosuppression monitoring and TAC dynamic adjustment models.

**Methods:**

This narrative review summarizes the benefits of ML for tacrolimus dosing in liver and kidney transplantation. Its key strength is integrating patients’ multidimensional clinical data to enable personalized dosing. We focus on ML applications in initial tacrolimus dose selection, early post-transplant concentration range determination, and dosage form conversion. We also systematically discuss current model limitations and future directions.

**Conclusion:**

ML has been widely applied to develop precise models for TAC immunosuppressive drug administration in patients after liver and kidney transplantation, providing a reference for individualized treatment. However, such models have not yet been widely applied in clinical practice and generally lack the ability to dynamically regulate drug concentrations over the long term. How to promote the model’s true application in clinical practice remains to be explored in depth, but it holds significant prospects for application and research value in individualized treatment.

## 1 Introduction

Immunosuppressive therapy is central to long-term anti-rejection management after liver and kidney transplantation (1). Tacrolimus (TAC), a potent calcineurin inhibitor, inhibits T lymphocyte activation and proliferation. Over 90% of patients worldwide receive TAC-based immunosuppressive regimens after transplantation (2, 3). However, the therapeutic window for TAC is narrow, and there is significant interpatient variability (IPV) (4). Even at the same blood drug concentration, individual pharmacokinetic (PK) differences (5)can lead to two markedly different outcomes: excessive concentrations can cause adverse reactions such as infection, hypertension, and hyperglycemia, and may increase the risk of neurotoxicity and nephrotoxicity (6–8); insufficient concentrations can trigger subclinical rejection and subsequently lead to progressive loss of graft function (9, 10).

**Figure 1:**
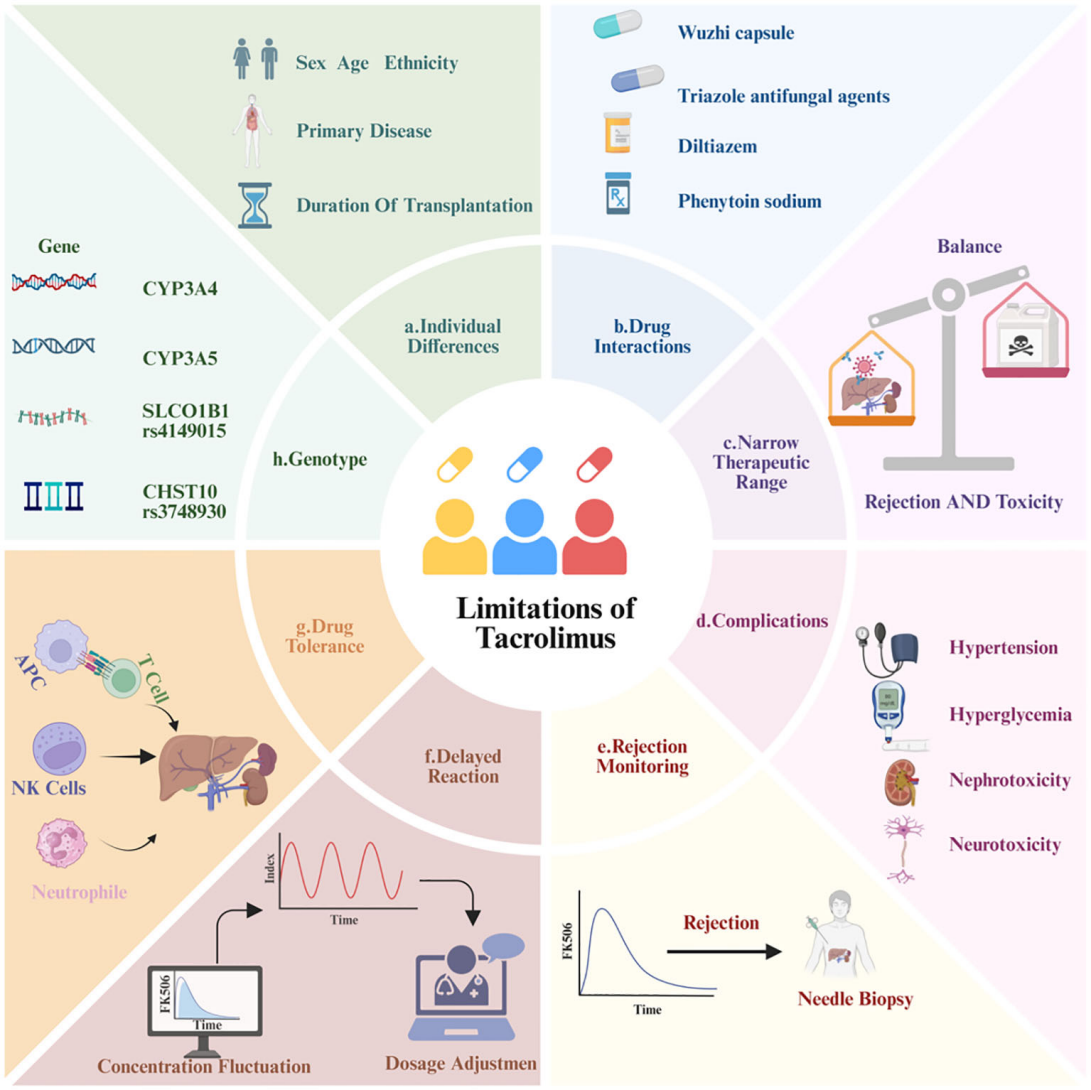
The key factors influencing the adjustment of tacrolimus include. **(A)** Individual differences such as gender, primary disease, and time after transplantation. **(B)** Drug interactions such as Wuzhi capsule, Triazole antifungal agents, Diltiazem, Phenytoin sodium, etc. **(C)** Narrow therapeutic range: excessive concentration causes toxicity, while too low a concentration leads to rejection. **(D)** Adverse reactions such as nephrotoxicity, neurotoxicity, hypertension, and hyperglycemia. **(E)** Pathological biopsy is the gold standard for diagnosing rejection reactions. **(F)** Delayed monitoring: there is a certain lag between concentration changes and liver/kidney function responses. **(G)** Antigen-presenting cells (APCs) and natural killer cells (NK cells), etc., lead to immune tolerance. **(H)** Gene polymorphisms (such as CYP3A4, CYP3A5, SLCO1B1 rs4149015, rs3748930, CHST10). Figures was created with BioRender.com.

There is a nonlinear relationship between the dose of TAC and blood drug concentration, and patients with high IPV have a significantly increased risk of rejection and complications (11). In clinical practice, dosing is typically adjusted based on TAC concentration monitoring and clinical response (12). However, this approach is empirical and time-lagged, making it difficult to optimize the medication plan in real time. As shown in **Figure 1**, TAC concentration is affected by numerous complex and interacting factors, which makes precise clinical regulation extremely challenging and calls for further improvements in safety and therapeutic efficiency. Machine Learning (ML), with its powerful capabilities in multi-dimensional data integration and nonlinear relationship mining, predicts the initial TAC dose and early post-transplant blood drug concentration, enabling a transformation from “empirical drug administration” to “precise prediction”. This article summarizes the progress of ML applications in TAC dose adjustment and concentration optimization and discusses the inherent limitations of existing models regarding data heterogeneity, clinical extrapolation, and real-time adaptability. On this basis, in response to the core demands of long-term management after transplantation, the direction and development potential of model construction with dynamic concentration-interaction characteristics were further prospectively explored.

## 2 Application of machine learning in transplantation

**Figure 2:**
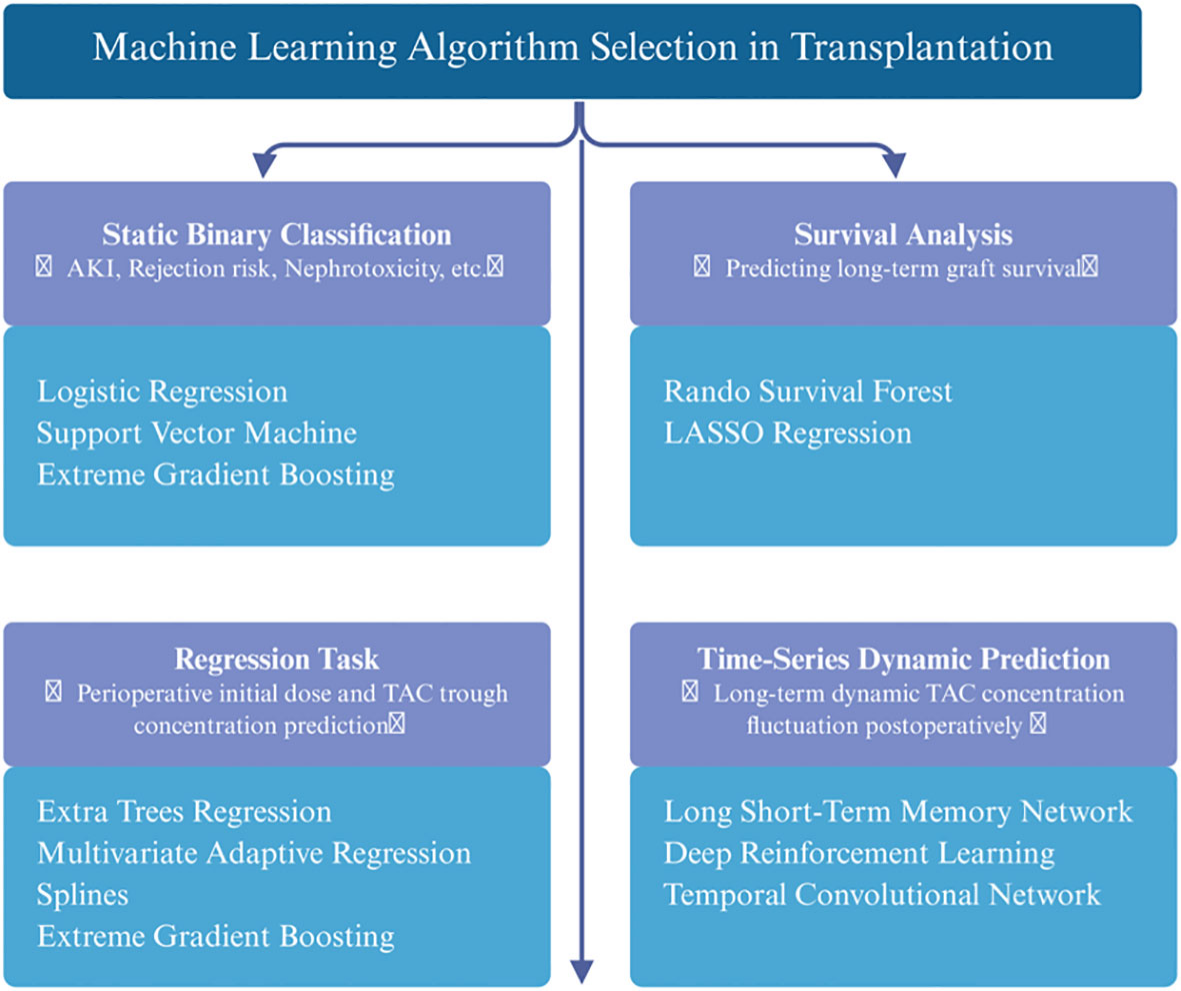
Machine learning algorithm selection in transplantation. Static binary classification (Logistic Regression, Support Vector Machine, Extreme Gradient Boosting) for predicting the risk of rejection and tacrolimus toxicity; Survival analysis (Random survival forest, LASSO regression) was used for the long-term prognosis of transplantation; regression model for tacrolimus dose and trough concentration prediction (Extra Trees regression, Multivariate Adaptive regression Splines, Extreme Gradient Boosting); And time series architectures (Long Short-Term Memory Network, Deep Reinforcement Learning, Temporal Convolutional Network) are used for long-term dynamic monitoring of tacrolimus blood exposure (56–58, 62). Figures was created with BioRender.com.

**Figure 3:**
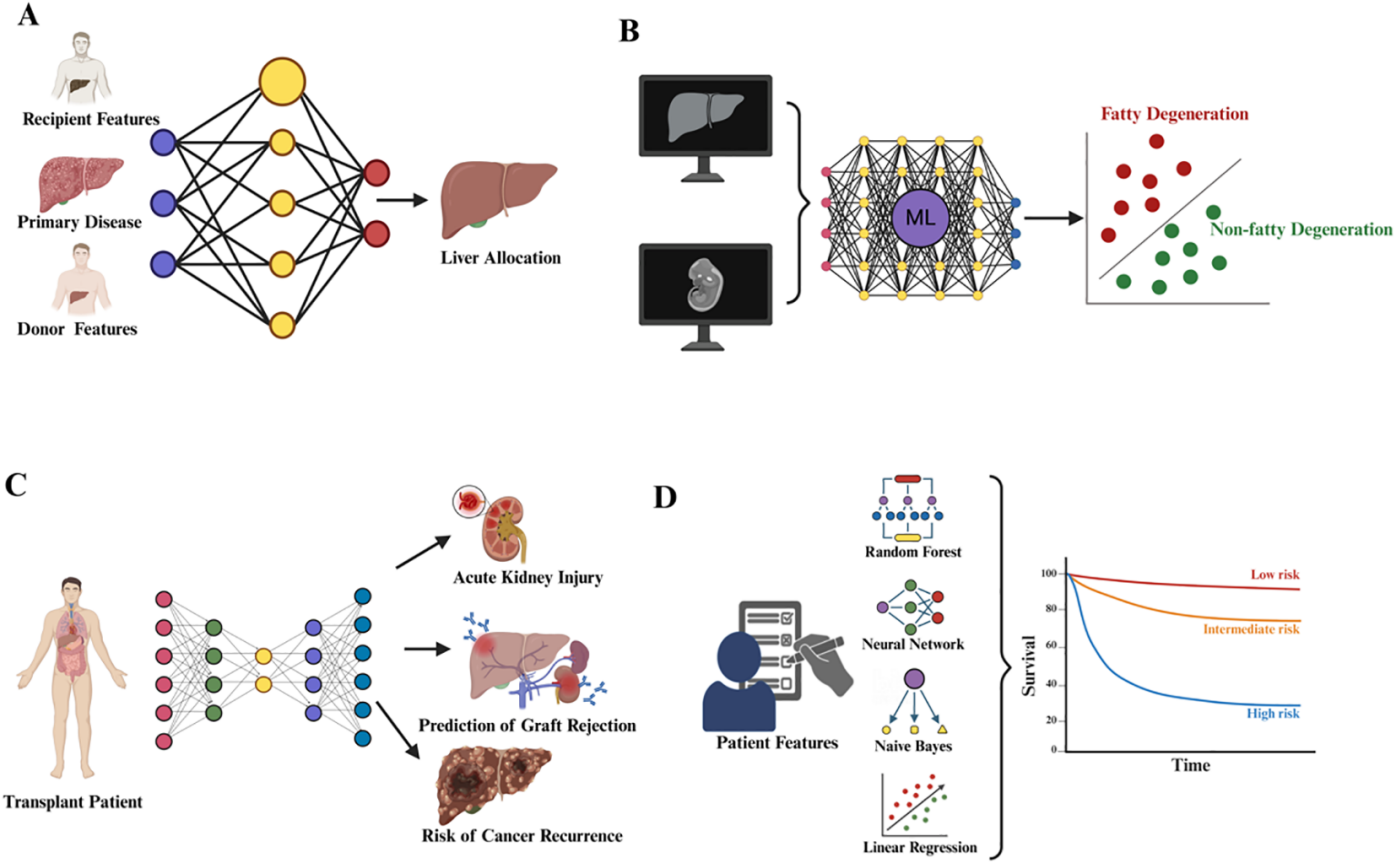
**(A)** Machine learning-driven donor-recipient matching for liver allocation: Neural networks integrate recipient characteristics, primary disease status, and donor features to optimize liver allocation decisions. **(B)** Multimodal imaging analysis for donor liver assessment: Machine learning models process liver and kidney imaging data to classify donor livers into fatty degeneration and non-fatty degeneration categories. **(C)** Prediction of post-transplant clinical outcomes: Neural networks analyze transplant patient data to predict risks of acute kidney injury, graft rejection, and cancer recurrence. **(D)** multi-algorithm graft survival prediction: Multiple machine learning methods (Random Forest, Neural Network, Naive Bayes, Linear Regression) are applied to patient features to stratify graft survival into low, intermediate, and high-risk groups. Figures was created with BioRender.com.

ML possesses powerful data mining and pattern recognition capabilities, capable of extracting correlations between model learning features and clinical goals from large volumes of clinical data, supporting decision-making and enhancing decision-making efficiency and accuracy (13, 14). Various ML algorithms have been widely applied in the clinical management throughout the entire transplantation process. **Figure 2** presents a dedicated algorithm selection framework developed based on the core clinical prediction tasks in liver and kidney transplantation. (**Figure 2**). Common ML methods include logistic regression (LR), support vector machine (SVM), random forest (RF), decision trees, gradient boosting trees, and artificial neural networks (ANN) (15–17). The application of ML in organ transplantation has spanned multiple processes, from preoperative assessment to postoperative management, with advantages in pre-transplantation risk assessment, donor-recipient matching, and post-transplantation prognosis prediction (**Figure 3**).

The scoring systems including SA10, L-Graft, and iBox established based on traditional linear regression and PK models have achieved favorable clinical performance and demonstrated unique advantages in predicting short−term patient survival, graft survival, and long−term prognosis, respectively (18–25). Though conventional linear scoring models have clinical value, ML models tend to support more comprehensive analysis and may see wider use in peri-transplant management. For instance, in simulated allocation, the optimal decision tree model can rank liver transplantation candidates more accurately and objectively based on disease severity, optimize the efficiency of liver source allocation, and effectively reduce the risk of death for patients during the waiting period for transplantation (26, 27); ANN can integrate the multidimensional characteristics of donors and recipients, thereby optimizing and improving the precision of the graft allocation plan (28, 29). Additionally, the RF model enables effective prediction of postoperative acute kidney injury (30). Meanwhile, ML can integrate multimodal images and digital pathological information to quantitatively assess the degree of steatosis in the donor liver, fibrosis in the donor kidney, etc., providing key references for organ quality assessment (31–33). The application summary of ML before and after transplantation is shown in **Table 1**. However, none of the above-mentioned models have been officially put into clinical application. Nevertheless, the core ability of ML to integrate multidimensional data has been fully demonstrated, providing an important direction for the development of precision medicine in transplantation.

**Table 1:** The application of machine learning in transplantation.

Author and year	Patient group	Machine learning method(s) used	Factors included	Key outcomes	References
Bertsimas et al., 2019	Liver transplant candidates	Optimal Classification Trees	MELD score Bilirubin, Age, Waiting time	OPOM model outperformed MELD in predicting 3-month outcomes mortality/removal.	(26)
Briceño et al., 2014	1003 liver transplant Recipients	Artificial Neural Networks	Donor age, BMI, MELD score, Hepatitis status Recipient age, BMI, MELD score Hepatitis status	The nN-CCR and NN-MS models of artificial intelligence neural networks predict that the 3-month graft survival rate of liver transplantation is significantly better than that of scores such as MELD, and they serve as powerful decision-making tools for optimizing donor-recipient matching.	(28)
Arisar et al., 2023	254 liver transplant recipients	Least Absolute Shrinkage Selection Operator	Donor age, Recipient age, Recurrence of primary etiology, TAC use, APRI score, Liver enzymes, Maximum liver attenuation from CT scans	The development of a radiomics model combined with clinical data shows relatively high accuracy in predicting graft fibrosis of grade ≥F2.	(6)
He et al., 2021	493 liver transplant Recipients	Random Forest Support Vector Machine Classical Decision Tree Conditional Inference Tree	Hepatic encephalopathy, Graft liver weight, Donor total bilirubin Intraoperative blood loss, Operative duration, Recipient BMI, MELD score, Serum creatinine, Albumin	The random forest model performs well in predicting the incidence of acute kidney injury after liver transplantation, with an accuracy of 0.79 and an AUC of 0.850, which is better than other machine learning models and logistic regression.	(30)
Fayzullin et al., 2024	120 patients with kidney transplant rejection	Mask Region-based Convolutional Neural Network	Pathological tissue Fibrosis Pathological Interstitial Infiltration	High precision in predicting clinical scores, potential for diagnosing acute kidney transplant rejection, robust AI model performance across datasets, and demonstrated high AUC for predicting rejection severity.	(32)
Jo et al., 2023	987 kidney transplant recipients	Random Forest Elastic Net Extreme Gradient Boosting	Recipient age, Donor BMI, ABO incompatibility, Donor HLA II mismatch, ATG induction	The incidence of subclinical rejection within 2 weeks after kidney transplantation was 14.6%. HLA II mismatch was identified as a risk factor, while ATG induction served as a protective factor. Logistic regression and elastic network machine learning models effectively predicted this reaction.	(49)

## 3 Machine learning-based initial dosage recommendation model for tacrolimus

### 3.1 Limitations of traditional pharmacokinetic and statistical models for TAC initial dosing

The initial dose of TAC administered to transplant patients after surgery is often calculated based on the patient’s weight (0.1-0.2 mg/kg), but studies have shown that only 37.4% of patients reach the target blood drug concentration on the third day after surgery, and an adjustment period of up to 3 weeks is often required (34). The blood drug concentration of TAC is influenced by multiple factors, including PK, gene polymorphisms, physiological factors, and drug interactions. Meanwhile, lifestyle factors such as dietary structure and sleep habits also indirectly affect the stability of its blood drug concentration (35–38). To address these drawbacks, Birnbaum et al. (34) pioneered and validated a conventional Population Pharmacokinetic (PopPK)-based dosing formula incorporating genetic and clinical factors for kidney transplant recipients, which systematically verified the core predictive variables affecting TAC clearance (39). Subsequent traditional modeling studies further refined TAC dosing strategies. For instance, traditional models have incorporated body surface area to optimize conventional weight-based dosing, correcting dose biases caused by abnormal body fat ratios and establishing genotype-stratified dosing strategies for CYP3A variants (40). This initial dose recommendation strategy has been validated in a small prospective trial involving 60 patients (41). Subsequent studies further integrated paired donor-recipient genotypic data to comprehensively characterize individual TAC metabolic phenotypes, compensating for the limitations of recipient-only genetic analysis (42). Researchers additionally incorporated dynamic perioperative clinical indicators and multiple TAC-metabolism-modifying concomitant medications, including Wuzhi capsules, voriconazole, and proton pump inhibitors, into PopPK models, further strengthening model predictive performance (43–45). Compared with empirical dosing strategies, traditional models integrating genotypic and clinical information significantly increased the 24-hour target TAC concentration attainment rate and reduced the incidence of new-onset metabolic syndrome (46). Furthermore, distinct nonlinear PK profiles of TAC have been identified between liver and kidney transplant populations. Based on these differences, conventional models have established scenario-specific dosing protocols tailored to genotypes, antifungal combination therapy, and abnormal hematological and liver function parameters (47). **Table 2** summarizes the above traditional PopPK and statistical models for TAC initial dose optimization.

**Table 2:** Traditional model−based initial dosage recommendation models for tacrolimus.

Author and year	Patient group	Modeling method	Factors included	Key outcomes	References
Birnbaum et al., 2011	681 kidney transplant Recipients	Nonlinear Mixed-Effects Model	Recipient CYP3A5 genotype Postoperative Day Steroid Drugs, Age, CCB use	The first tacrolimus dosing model that combines genetic and clinical factors predicts CL/F to determine the dose for TAC concentration.	(34)
Louise M. Andrews et al., 2019	337 kidney transplant Recipients	Nonlinear Mixed-Effects Model	CYP3A5 genotype CYP3A4 genotype Age BSA	Combining CYP3A4/CYP3A5 genotypes, age, and BSA lowers the risk of sub- or supratherapeutic levels, surpassing traditional weight-based dosing.	(40)
Cai et al., 2022	176 Adult liver Transplant Recipients	Nonlinear Mixed-Effects Model	Postoperative Day Hematocrit, Total Bilirubin CYP3A5 genotype Triazole Antifungal	The Michaelis-Menten (MM) model outperformed the theory-based linear model in predicting TAC dosing.	(47)
Yi Wu et al., 2021	210 liver transplant Recipients	Multivariate Linear R	Daily Dose of TAC Donor and recipient CYP3A5 genotype (rs776746) Recipient SLCO1B1 genotype(rs2291075) Total Bilirubin	The SLCO1B1 rs2291075 polymorphism was linked to TAC metabolism, influencing dose-adjusted trough concentrations (C/D) during the early postoperative period.	(42)
Shi B et al., 2023	150 liver transplant Recipients	Nonlinear Mixed-Effects Model	Weight, Total bilirubin, Donor and recipient CYP3A5 genotype (rs776746) Recipient SLCO1B1 genotype (rs4149015) and CHST10 genotype(rs3748930)	The genotype-based initial dosing model greatly enhances target attainment and decreases the need for dosage modifications.	(46)

Despite these iterative optimizations, conventional PopPK and statistical models are constrained by inherent limitations. Restricted by preset linear or semi-mechanistic assumptions, they fail to capture high-dimensional, nonlinear interactions among complex clinical variables and lack sufficient capacity for multi-source data fusion and dynamic dose correction. During dose regulation, both low and high blood concentrations of TAC can damage the transplanted organ (48, 49). The traditional dose adjustment method based on clinical experience has a clear lag (40, 50). Identifying patients at risk of abnormal TAC concentrations in advance has become an increasingly important direction for individualized and precise regulation of immune strategies (51).ML can integrate multidimensional clinical data, automatically screen key dosing-influencing factors, and quantify intricate dose-concentration relationships, enabling precise individualized TAC dosing stratification and clinical optimization (52). It can not only accurately identify the key features that affect the concentration of TAC but also construct models to quantitatively present the complex nonlinear relationships among various factors.

### 3.2 Machine learning-driven precision individualized dosing for perioperative TAC

ML can accurately associate TAC dose with outcomes by characterizing the complex nonlinear relationship between them. Among multiple ML algorithms, the optimal Regression Tree algorithm performs best in predicting the stable dose of TAC (14). By integrating multidimensional variables and incorporating gene-drug interaction factors, ML can provide a more refined basis for individualized TAC dose adjustment and significantly improve prediction accuracy (53). In specific clinical scenarios involving laboratory test data from liver transplant patients collected 14 days after surgery, the Long Short-Term Memory Network was compared with the LR and PopPK models. The results showed that the Long Short-Term Memory Network’s dose recommendations effectively increased the rate of reaching the TAC target concentration, reduced the risk of transplant rejection, and shortened intensive care unit length of stay (54). In addition, when the model incorporates concomitant medications such as Wuzhi capsule and nifedipine, along with other key factors affecting TAC concentration, such as genotype, the efficacy of the individualized dose-prediction model for TAC during the perioperative period can be further optimized (55). However, the selection of the optimal ML algorithm varies across studies: another study comparing 10 ML algorithms found that the extreme Random Forest Regression algorithm had the best predictive performance and could effectively improve the accuracy of individualized TAC dose adjustment during the perioperative period. A daily dose prediction model for TAC was constructed using clinical data collected within 3 months after kidney transplantation. It was confirmed that the TabNet algorithm achieved the best predictive performance, providing a reliable reference for accurately predicting TAC dose in the early post-transplantation period (52). Among kidney transplant patients who used TAC for ≥1 year, after analyzing the factors affecting the stable dose and adverse reactions of TAC through six ML algorithms, including Generalized Linear Model, SVM, and ANN, it was found that the Generalized Linear Model was the optimal model for predicting the stable dose of TAC. Among them, the POR*28 and ABCB1 genotypes are the core predictors (56). In addition to genotype, incorporating hematocrit and CYP3A5 genotype into the PopPK model, combined with Extreme Gradient Boosting (XGBoost), has been shown to improve the model’s efficacy in predicting TAC trough concentrations in both liver and kidney transplantation clinical scenarios (51, 57).

With the gradual prolongation of transplant patients’ survival time, precise regulation of TAC is of great significance for protecting graft function while reducing drug toxicity and side effects. A single-center study in Japan (58) used ML to predict nephrotoxicity in patients with autoimmune diseases treated with TAC, and the results showed that SVM performed best. Although this study did not involve organ transplant patients, it provided a reference for predicting TAC-related adverse reactions in organ transplant patients with ML. In addition, given the unique clinical characteristics of immune tolerance in transplant patients, dose adjustment should be closely tailored to each patient’s tolerance potential (59). Indicators such as age at transplantation, CYP3A5 genotype, transplantation type, postoperative massive ascites, preoperative prothrombin time, international normalized ratio, creatinine, and total bilirubin have been confirmed as key features for identifying immune tolerance status. For patients with high immune tolerance, the dosage of TAC can be appropriately reduced to avoid the risks brought by excessive immunosuppression. At the same time, it is necessary to avoid blindly reducing the dosage for patients with low immune tolerance to prevent the rejection reaction caused thereby (60). The ML methods applied in the above model and the relevant features included are shown in **Table 3**.

**Table 3:** Predict the dynamic changes of tacrolimus based on machine learning.

Author and year	Patient group	Machine learning method(s) used	Factors included	Key outcomes	References
Tang et al., 2017	1045 kidney transplant Recipients	Artificial Neural Network Regression Tree Multivariate Adaptive Regression Splines Boosted Regression Trees Support Vector Regression Random Forest Regression Lasso Regression Bayesian Additive Regression Trees	Hypertension Diabetes Omeprazole use CYP3A5 genotype	The optimal model is the RT, whose MAE is similar to that of the linear model, and the clinical ideal rate is 4% higher than that of the traditional model.	(14)
Zhang et al., 2022	584 kidney transplant Recipients	Tabular Data Network Extreme Gradient Boosting Light Gradient Boosting Machine Categorical Boosting Gradient Boosting Decision Tree Random Forest, Support Vector Regression, K-Nearest Neighbors, Least Absolute Shrinkage and Selection Operator, Ridge Regression	Last TAC daily dose Tacrolimus TDM Postoperative Day Hematocrit, Serum creatinine, AST Weight, BMI, Uric acid CYP3A5 genotype	TabNet model yielded the best overall predictive performance, with R2 = 0.824, MAE = 0.468 mg/d, and a dose prediction accuracy of 86.19% within the ±20% error range.	(52)
Sridharan et al., 2023	120 Adult kidney transplant Recipients	Generalized Linear Model Support Vector Machine Artificial Neural Network Chi-square Automatic Interaction Detection Classification and Regression Tree K-Nearest Neighbors	Age, Sex ABCB1 genotype POR*28 genotype CYP3A5 genotype	For predicting the rate of TAV concentration within the therapeutic range, ML model outperformed traditional models. The MAE dropped from 17.7 to 16.4 and RMSE from 24.2% to 21.2%.	(56)
Fu et al., 2022	2551 kidney transplant perioperative Recipients	Adaptive Boosting Regression Extra Trees Regression Random Forest Regression Least Absolute Least Squares Multivariate Adaptive Regression Splines K-Nearest Neighbors Regression Support Vector Regression Multi-Layer Perceptron Regression Decision Tree Regression	CYP3A5 genotype(rs776746) CYP3A4 genotype(rs4646437) Hematocrit Wuzhi capsules TAC daily dose Age, Weight, Height Postoperative time Nifedipine, Medication history	The ETR performed the best. The proportion of test set R2 = 0.85 and dose deviation ≤20% was 92.77%, and the overall prediction accuracy reached 97.73%.	(55)
Wang et al., 2024	127 kidney transplant Recipients	Multi-Layer Perceptron Support Vector Machine Extreme Gradient Boosting	Hematocrit, RBC, Albumin, Bilirubin, Creatinine clearance CYP3A5 genotype Weight, Postoperative day Concomitant medications	The PopPK based XGBoost model yielded the lowest MAE = 1.61, MAPE = 23.7% and RMSE = 2.03 on the test set, outperforming the PopPK model across all metrics.	(51)
Li et al., 2023	145 liver transplant Recipients	Extreme Gradient Boosting	TAC daily dose, Postoperative days Voriconazole CYP3A4 genotype CYP3A53 genotype Weight, Age, Sex, Hematocrit, Albumin, Bilirubin	The XGBoost model yielded a lower MPE for TAC trough concentration than the conventional PopPK model.	(57)
Yoon et al., 2024	443 liver transplant Recipients	Long Short-Term Memory Gradient Boosting Regression Trees	TAC dose, Concentration Weight, Height, ALT, AST Total bilirubin, INR, Albumin Creatinine, Hematocrit Age, Sex	Internal and external validation confirmed that the LSTM model outperformed GBRT, LR and PopPK models, with MDPE = 8.8%, RMSE = 1.7 ng/mL and MAE = 1.5 ng/mL.	(54)

### 3.3 Pharmacokinetic and immunological differences between liver and kidney transplant recipients

TAC-based immunosuppression is widely used in both liver and kidney transplantation, yet the two recipient groups differ in TAC PK, therapeutic targets and post-transplant immune status. Allogeneic liver grafts express donor-derived CYP3A4/CYP3A5 in hepatocytes, which dominate TAC clearance in liver transplant patients. This donor gene-related metabolic mechanism does not exist in kidney transplant recipients (4). Due to divergent early post-transplant PK, liver transplant recipients receive lower target trough concentrations to reduce nephrotoxicity, while higher early trough levels are used in kidney transplant recipients to avoid acute rejection (50). General ML models for combined cohorts tend to perform poorly when applied to either group alone. For accurate and clinically applicable ML models for TAC dosing, strict subgroup stratification is necessary. Donor genetic features should be emphasized in liver transplant models, and kidney function indices as well as recipient genotypes in kidney transplant models. In view of different TAC fluctuation patterns, targeted algorithms are recommended: time series algorithms fit liver transplant cohorts with unstable drug levels, and traditional tree-based algorithms including XGBoost and RF are more suitable for kidney transplant patients with stable PK.

## 4 Varied performance of machine learning models for TAC prediction

These models provide clinicians with guidance on optimizing TAC dosing. Although the studies mentioned above also used ML methods, the conclusions reached varied. The core reason is that different ML models have distinct advantages for predicting TAC dosing. These discrepancies are further driven by sample size, feature dimensionality and population characteristics when selecting ML algorithms. RF fit single-center transplant cohorts with limited samples, whereas LR is ideal for datasets with sufficient samples and few features. SVM suits high-dimensional sparse data, while ANN require large datasets to function effectively (15). Because research samples, populations, and included variables differ, there is no unified standard for selecting the optimal algorithm, making it difficult to establish consistent clinical application norms. Although ML has been applied to developing TAC blood drug concentration prediction models, these models have not yet achieved clinical translation or practical application, making it difficult to meet the actual needs of immunosuppression management after transplantation. On the one hand, existing models are mostly built on small-sample retrospective data. They not only fail to incorporate long-term follow-up data from transplant patients but also lack external validation from large-sample prospective cohorts, making it difficult to ensure the models’ generalizability and long-term stability. In clinical settings, transplant recipients usually receive combined immunosuppressive therapies including everolimus, sirolimus, mycophenolate mofetil and glucocorticoids. Current models only predict tacrolimus levels while neglecting drug-drug interactions, failing to reflect real-world immunosuppressive regimens. Additionally, these models do not incorporate dynamic changes in immune function and metabolism over time, nor do they link TAC concentrations to clinical outcomes such as rejection, nephrotoxicity and infection, limiting their value for individualized treatment.

## 5 Machine learning-based switch between immediate-release and prolonged-release tacrolimus

In addition to dose adjustment research on the common immediate-release tacrolimus (IR-T), studies are also exploring dose optimization for the sustained-release, prolonged-release tacrolimus (PR-T). The precise drug administration strategies for different dosage forms are summarized in **Table 4**. Multiple adaptive regression splines can accurately predict the Area Under the Curve (AUC) of PR-T based on two TAC blood drug concentration points at 0h and 12h, providing a reliable basis for clinicians to adjust the dosage (61). However, this approach requires relatively precise sampling times. The XGBoost model can estimate AUC after IR-T and PR-T administration using 2–3 TAC concentration points and key covariates, such as the administration dose (12). Although this model has been verified in the training of the Monte Carlo simulation dataset (62), its development and validation are primarily based on data from kidney transplant patients, which may introduce bias for liver transplant patients. In addition, there is inevitably a gap between simulated data and real-world patient data in clinical scenarios, and it is impossible to fully reflect individual differences in real-world diagnosis and treatment. The CYP3A4/CYP3A5 genotype plays a key role in epigenetic clearance in both PR-T and IR-T. However, the ABCB1 transporter gene polymorphism, which significantly affects the blood drug concentration of IR-T, has no significant effect on PR-T (63). The research suggests that this is due to the slow release of PR-T preparations and the concentration of absorption sites in the distal small intestine, which weakens the “pumping effect” of ABCB1 on drug absorption, thereby reducing the influence of this gene polymorphism on drug absorption. In addition, due to large fluctuations in IR-T blood concentrations, some patients may experience adverse reactions in the nervous or digestive systems. Therefore, for such patients, it is usually considered to switch to PR-T to maintain stable blood drug concentrations, thereby reducing the occurrence of related side effects. A prospective randomized crossover study among African Americans in the United States found that the peak concentration of IR-T in CYP3A5 expressors was 33% higher than that in non-expressors, while the bioavailability of PR-T was 32% higher than that of IR-T. Therefore, during drug conversion, to ensure the consistency of drug exposure, it is usually necessary to reduce the dose of IR-T by 20% to convert to PR-T (64). In a comparison of the PK characteristics of IR-T and PR-T using the PopPK model, the results showed that the relative bioavailability of the two preparations was similar, and the trough concentrations were comparable, supporting the clinical dose conversion of 1mg:1mg (65). The differences in the conclusions of the two studies mainly stem from the significant impact of race on TAC bioavailability (66).

**Table 4:** Applications of machine learning and traditional models in conversion between immediate-release and prolonged-release tacrolimus.

Author and year	Patient group	Modeling method	Factors included	Key outcomes	References
Woillard et al., 2020	2835 kidney, liver, and heart transplant Recipients	Extreme Gradient Boosting	TAC concentrations, Dose Transplant type Age, Postoperative Day	XGBoost models for tacrolimus AUC prediction demonstrated excellent performance, with bias under 5% and RMSE below 10%, surpassing MAP Bayesian estimation.	(62)
Ponthier et al., 2023	113 liver transplant Recipients and 97 kidney transplant Recipients	Multivariate Adaptive Regression Splines, Extreme Gradient Boosting Generalized Linear Model with Elastic Net Regularization	Blood concentrations Age, Sex, Transplant type Hematocrit	Multivariate Adaptive Regression Splines can precisely predict the AUC of PT-T based on the 0h and 12h TAC blood concentration points.	(61)
Mohammed Ali et al., 2023	98 kidney transplant Recipients	Population Pharmacokinetic	CYP3A5 genotype CYP3A4 genotype ABCB1 genotype Age, Sex, BMI, Weight Hematocrit	By incorporating CYP3A4/CYP3A5 polymorphic metabolic clusters, a population pharmacokinetic model for LCP-Tac tacrolimus was created in stable kidney transplant patients.	(63)
Jennifer et al., 2017	50 African American kidney transplant Recipients	Mixed Effects Analysis of Covariance Model	CYP3A5 genotype 24-hour tacrolimus pharmacokinetic profiles (AUC, Cmax, Cmin, Tmax, bioavailability)	IR-T and PR-T differed substantially in the PK of TAC. While CYP3A5 genotype influenced drug kinetics, PR-T had better bioavailability and was less affected by this genotype.	(64)
Lu et al., 2019	408 liver, kidney, and heart transplant Recipients	Population Pharmacokinetic	Age, Sex, Race, Weight, AST, ALB Organ transplanted type	Developing a population pharmacokinetic model for tacrolimus, showing similar PK profiles between IR-T and PR-T formulations. Differences in clearance and bioavailability across races support a 1mg:1mg clinical conversion.	(65)

This result examined the influence of race on individual differences in the PK of TAC and provided a new idea for dose conversion between IR-T and PR-T, which is conducive to improving the accuracy of individualized medication. Although most current studies still support the 1mg:1mg dose conversion regimen for IR-T and PR-T, there is no clear consensus or unified clinical guideline on the core clinical issue of “when to switch between the two preparations”. Models trained on East Asian cohorts may yield biased predictions for European, African and other ethnic populations. External validation across ethnicities requires multi-population clinical and genetic data as well as model parameter recalibration.

## 6 Summary and outlook

### 6.1 The progress and limitations of existing machine learning models

Research on ML for personalized tacrolimus dosing has advanced in data expansion, algorithm optimization, and clinical adaptation, showing strong application potential. How to effectively transform the constructed model into a clinically applicable tool is the core issue that all related research must confront directly in the modeling process. Current TAC prediction models are mostly developed based on small-sized cohorts of Chinese populations, which frequently leads to overfitting and poor reproducibility. Future large-scale data collection requires strict multicenter standardization for data preprocessing, including missing value imputation, outlier removal and feature normalization, to eliminate systematic bias in raw data. Rigorous cross-validation is also essential to objectively evaluate model performance and reduce sampling bias, thereby improving the generalization ability and reproducibility of ML models for TAC prediction. Notably, ML models established in a single ethnic group require targeted revalidation and parameter optimization before application to other populations.

Transplant recipients generally present with complex clinical conditions, and individual heterogeneity in the type and severity of complications further restricts the generalizability of the model. Additionally, liver and kidney transplant patients differ intrinsically in physiological status, organ functional demands and postoperative immune responses. Accordingly, more refined subgroup stratification is required during model development to improve its applicability. In clinical practice, transplant recipients often receive combination immunosuppressive regimens centered on TAC. Routine therapeutic drug monitoring primarily focuses on TAC, with limited continuous concentration data for mycophenolate mofetil and glucocorticoids. Additionally, distinct immunosuppressive mechanisms hinder the conversion of doses across different immunosuppressants into a unified intensity index. Given these constraints, we propose phased strategies for future model development. To accommodate combination therapy in prediction models, future studies should incorporate other commonly used immunosuppressants (e.g., mycophenolate mofetil, glucocorticoids, and calcineurin inhibitors) as key covariates. On this basis, ML can be used to screen and recommend the best combination regimens for different patient subgroups. The quantitative correlation analysis between drug dosage and overall immunosuppression intensity can be further clarified on this basis through large sample calculation and comprehensive subgroup analysis. ML can then be applied to identify optimal combination strategies for populations with different clinical characteristics, thereby providing TAC concentration recommendations that better reflect clinical scenarios.

### 6.2 Clinical application and developmental prospects of machine learning

To be widely adopted clinically, the model needs standardized external validation and assessment. Apart from multicenter perspective external validation in independent cohorts, the model’s discrimination, accuracy and calibration should be evaluated in subgroups of different transplant types, ethnicities, ages and combined medications. An AUC-ROC greater than 0.70 suggests basic clinical applicability, and a value exceeding 0.80 signifies superior performance. Mean Absolute Error (MAE) and Root Mean Square Error (RMSE) are also used to assess overall prediction capacity. For models with unsatisfactory calibration, improvements can be made through parameter tuning, feature optimization, and professional recalibration algorithms. On this basis, clinical simulation decision-making and real-world application research are carried out. The dose and concentration suggestions output by the model are embedded into the clinical workflow, and the transplant physicians independently evaluate the interpretability, operability and clinical consistency of the suggestions. At the same time, a mechanism for collecting physician feedback and iterating the model should be established. The feature weights, decision thresholds and output forms should be continuously optimized in combination with clinical practice to make the model recommendations more in line with real diagnosis and treatment habits.

In terms of model design, the current ML models’ recommendations for TAC dosage or blood drug concentration are largely limited to analyzing the impact of static patient characteristics on drug concentration and then providing a single medication reference. However, during long-term follow-up after transplantation, changes in T cell subsets, regulatory T cell function and inflammatory mediators drive continuous shifts in the patient’s immune status, accompanied by the gradual development of immune tolerance. Current ML models cannot fully characterize this dynamic immune regulation process. The required immunosuppressive intensity also shows phased adjustment needs. The static recommendation model is clearly difficult to match the actual dynamic diagnosis and treatment needs in clinical practice. Therefore, the introduction of dynamic time series data and specialized models such as long short-term memory, temporal convolutional networks, and Deep Q-networks may better adapt to clinical dynamic regulation. By collecting long-term longitudinal data, including TAC concentration, laboratory indicators, immune parameters, dose changes and concurrent medication. The stability of the model was tested by using sliding window verification and phase evaluation, and the prediction accuracy of different subsequent stages was evaluated by using mean absolute percentage error (MAPE). Combining the time series framework with traditional ML can enable the model to integrate static baseline features and dynamic trends. By achieving the transformation from “static feature-driven single recommendation” to “dynamic time series data-driven precise adaptation”, the problem of dynamic regulation of immunosuppressive drugs in long-term clinical follow-up is solved. In the future, such models may be embedded in electronic medical record systems to automatically extract comprehensive patient data. With the continuous accumulation of multi-center longitudinal clinical data, the model can constantly self-learn and optimize, and further formulate real-time adjustable medication intervals and flexible, targeted immunosuppressive regimens for each patient. Of course, this requires a huge database support as well as more precise algorithm support.
