# Correction: Machine learning driven precision medicine in tacrolimus dosing: current research and future perspectives in liver and kidney transplantation

**DOI:** 10.3389/fimmu.2026.1946015

**Published:** 2026-07-31

**Authors:** Hanbei Lv, Zhangpeng Feng, Shusen Zheng, Guoping Jiang

**Affiliations:** 1 School of Medicine, Zhejiang Chinese Medical University, Hangzhou, China; 2 Department of Hepatobiliary Pancreatic Surgery, Key Laboratory of Artificial Organs and Computational Medicine in Zhejiang Province, Shulan (Hangzhou) Hospital, Shulan International Medical College, Zhejiang Shuren University, Hangzhou, China; 3 Department of Hepatobiliary Pancreatic Surgery, Shulan (Hangzhou) Hospital, Shulan International Medical College, Zhejiang Shuren University, Hangzhou, China

**Keywords:** blood concentration prediction, dynamic dose optimization, individualized treatment, machine learning, organ transplantation, tacrolimus

Affiliation “School of Medicine, Zhejiang Chinese Medical University, Hangzhou, China” was erroneously omitted for authors Hanbei Lv and Zhangpeng Feng. Affiliation “Department of Hepatobiliary Pancreatic Surgery, Shulan (Hangzhou) Hospital, Shulan International Medical College, Zhejiang Shuren University, Hangzhou, China” was omitted for authors Hanbei Lv, Zhangpeng Feng, Shusen Zheng and Guoping Jiang.

The original version of this article has been updated.

